# Defining Sudden Infant Death and Sudden Intrauterine Unexpected Death Syndromes with Regard to Anatomo-Pathological Examination

**DOI:** 10.3389/fped.2016.00103

**Published:** 2016-09-21

**Authors:** Giulia Ottaviani

**Affiliations:** ^1^Department of Biomedical, Surgical and Dental Sciences, “Lino Rossi” Research Center for the Study and Prevention of Unexpected Perinatal Death and SIDS, University of Milan, Milan, Italy

**Keywords:** sudden infant death syndrome, sudden intrauterine unexpected death syndrome, anatomo-pathological examination definition, cardiac conduction system, brainstem, autopsy

## Abstract

Crib death, or sudden infant death syndrome (SIDS), is the most frequent form of death in the first year of life, striking one baby in every 1,700–2,000. Yet, despite advances in maternal–infant care, sudden intrauterine unexplained/unexpected death syndrome (SIUDS) has a sixfold to eightfold greater incidence than that of SIDS. Frequent congenital abnormalities, likely morphological substrates for SIDS–SIUDS, were detected, mainly represented by alterations of the cardiac conduction system, such as accessory pathways and abnormal resorptive degeneration, and hypoplasia/agenesis of the vital brainstem structures. On the basis of these considerations, the new common definition of the SIDS–SIUDS complex is “The sudden death of a fetus after the 25th gestational week or infant under one year of age which is unexpected by history and remains unexplained after a thorough case investigation, including examination of the death scene, performance of a general autopsy and examination of the fetal adnexa”. Therefore, given that the general autopsy does not disclose any cause of death, a more in-depth histopathological analysis of the cardiac conduction system and autonomic nervous system by specialized pathologists is necessary.

## Introduction

Sudden infant death syndrome (SIDS), or crib death, is the most frequent form of death during the first year of life. The sudden unexpected death of a baby in the crib and, even worse, in the mother’s womb is surely one of the most heartbreaking tragedies for the family, the pathologists, clinicians, and epidemiologists, as well as the general public, given that the affected individuals, before the lethal event, were regarded as healthy.

According to the Centers of Disease Control and Prevention (CDC) (1), SIDS has a death rate of 0.42 per 1,000 births, striking one baby in every 1,700–2,000. In developed countries, one in 100–200 pregnancies ends in stillbirth, which has a sixfold to eightfold greater incidence than that of SIDS and remains completely unexpected in 40–80% of cases, occurring in pregnancies that had seemed problem-free. Unexpected stillbirth has a sixfold to eightfold greater incidence than that of SIDS. The frequency of sudden intrauterine unexplained/unexpected death syndrome (SIUDS), which has ranged from 5 to 12‰ in the last 25 years, has not declined significantly despite modern advances in maternal–infant care (2–4).

The consequences among families are devastating, with high social cost, considering the unexpected loss of many potentially productive individuals. Despite the increasing general interest and number of published works, SIDS and SIUDS represent a great enigma of modern medicine whose etiology remains uncertain.

According to the most recent report from the American Heart Association (5), congenital heart diseases occur in about 0.8% of full-term live births. The true incidence of congenital heart diseases could be significantly higher than the reported one, given that cardiac anomalies occur 10 times more frequently in stillborn and premature infants than in full-term infants. Anatomic cardiac anomalies and channelopathies are important considerations for congenital heart disease inclusion, being characterized by common congenital histopathological substrates (6).

Even if SIUDS and SIDS are generally considered diagnoses of exclusion, subtle developmental abnormalities of the cardiac conduction system and brainstem have been highlighted. Risk factors of SIDS and SIUDS include exposure to maternal smoking, air and water pollution, food contamination, agricultural and household pesticides, etc., that, starting *in utero*, act as causative and triggering factors in vulnerable infants with developmental abnormalities in the cardiac conduction system and/or autonomic nervous system. Specific environmental risk factors can interact in complex ways with the genetic constitution leading to polymorphisms and/or mutations of specific genes. At least four categories of genes are involved in the pathogenesis of SIDS and SIUDS: genes for ion channel proteins involved in cardiac channelopathies, mainly the sodium channel gene (*SCN5A*/*LQT3*) and the potassium channel genes (*KCNQ, KCNH12, KCNE2*); genes regulating the brainstem functions, such as serotonin transporter (*5-HTT*), the regulator of the synaptic serotonergic receptor binding in the brainstem, *PHOX2B*, the major gene involved in congenital central hypoventilation syndrome (CCHS), and *En*-2, involved in the development of the autonomic nervous system; genes regulating inflammation and infections, such polymorphisms in complement C4 and interleukins IL-6 and IL-10; genes regulating energy production, hypoglycemia, and thermal regulation, such as the medium-chain acyl CoA dehydrogenase (*MCAD*) genes (2, 7, 8).

The objective that this study wishes to achieve is to introduce a new definition for SIDS and SIUDS, based on the anatomo-pathological findings from the investigation of a wide number of cases, with regard to the common pathogenesis of SIDS–SIUDS. This increased knowledge would untimely lead to the development of targeted risk-lowering strategies to reduce the incidence of these deadly forms of death.

## Discussion

Over the years, increasing interest has been focused on the possible developmental anomalies of the cardiac conduction and autonomic nervous systems involved in the pathogenesis of SIDS and SIUDS. This interest has led to investigations of a wide number of fetuses and infants from the 25th gestational week through the first year of postnatal life (2, 9).

Retrospective research cases are currently represented by over 140 SIDS, 120 SIUDS victims, and 60 age-matched controls, since the Italian national authority (10) decreed that the regional and national cases of supposed SIDS or SIUDS were to be converged and investigated at the “Lino Rossi” Research Center of the University of Milan. All cases were accurately studied through analysis of clinical data, of the risk factors, and particularly *postmortem* through the histopathological investigation of the cardiac conduction system and of the autonomic nervous system, both central and peripheral (11). SIDS and SIUDS share developmental abnormalities of the cardiac conduction system, such as defective or exaggerated resorptive degeneration, atrioventricular dispersion or septation, conductive accessory pathways, cartilaginous meta/hyperplasia of the central fibrous body, and dualism of the atrioventricular junction, as morphological substrates for cardiac arrhythmias. SIDS and SIUDS also share developmental abnormalities of the vital brainstem structures, such as hypoplasia, agenesis or neuronal immaturity of the arcuate, hypoglossus, pre-Bötzinger, cochlear, locus coeruleus, or Kölliker-Fuse nuclei. Therefore, the need to perform an in-depth study of the structures of the cardiac conduction and of the autonomic nervous systems, modulating the respiratory, cardiovascular, upper-digestive, and arousal activities, is self-evident.

Genetic mutations underlying cardiac arrhythmias have been focused on the long-QT syndrome and have been detected in no more than 10% of SIDS and of unexplained intrauterine fetal deaths (12, 13). The genetic studies of increasing interest involve the application of advanced analytical approaches, including whole exome sequencing to cases of sudden unexpected death in neonates and infants (14, 15).

The new perspective of the SIDS and SIUDS definition includes the detection of common congenital anomalies of the cardiac conduction and autonomic nervous systems, similar in both SIDS and SIUDS victims, which indicate a continuity between these two deadly forms of death that can, therefore, be regarded as the so-called SIDS–SIUDS complex.

### Sudden Infant Death Syndrome

Currently, the CDC (16) describe SIDS as a subset of sudden unexpected infant death (SUID), defined as the “death of an infant less than one year of age that occurs suddenly and unexpectedly, and whose cause of death is not immediately obvious before investigation”. SUID combines the following three forms of death: SIDS, death by unknown cause, and accidental suffocation, and strangulation in bed (ASSB) (17).

In 1970, Beckwith (18) published the first definition of SIDS introduced at the second international conference held in Seattle, WA, USA, in 1969, as follows: “The sudden death of any infant or young child, which is unexpected by history, and in which a thorough postmortem examination fails to demonstrate an adequate cause for death”. This definition does not refer to specific age or to any common features and SIDS was only a syndrome of exclusion.

In 1991, Willinger et al. (19) published the following new definition of SIDS according to the National Institute of Child Health and Human Development (NICHD) panel convened in 1989: “The sudden death of an infant under one year of age, which remains unexplained after a thorough case investigation, including performance of a complete autopsy, examination of the death scene, and review of the clinical history”. SIDS becomes a diagnosis made by pathologists, based primarily on the finding of a negative autopsy. This definition has been largely adopted all over the world for over 20 years.

In 2002, Matturri et al. (20), at the 7th International Conference on SIDS, proposed that the definition of SIDS as “the sudden death of an infant under one year of age which remains unexplained after a thorough case investigation, including the performance of a complete autopsy, examination of the death scene, and review of the clinical history” should be modified by adding, at the end, the following: “a complete autopsy with an in-depth histopathologic analysis of the cardiorespiratory innervation and specialized myocardium, performed only by an experienced, reliable pathologist”.

In 2004, Krous et al. (21) defined SIDS as “The sudden unexpected death of an infant less than one year of age, with onset of the fatal episode apparently occurring during sleep, that remains unexplained after a thorough investigation, including performance of a complete autopsy and review of the circumstances of death and the clinical history”.

This latest definition of SIDS that describes SIDS as unexplained after a complete autopsy is, hereby, under review as the findings in the cardiac conduction and autonomic nervous systems detected in SIDS can be morphological substrates for the sudden unexpected death.

In 2016, Goldstein et al. (22) reported that there is no consensus on the use of the term SIDS, as external factors, such as prone sleep position or bed sharing, may at times explain the cause of death as positional asphyxia or accidental suffocation.

### Sudden Intrauterine Unexplained Death Syndrome

In 2001, Frøen et al. (23) defined sudden intrauterine unexplained death as the “Intrauterine death before the onset of labor of a fetus at ≥22 completed weeks of gestation or with ≥500 g body mass, which is unexpected by history and in which a thorough autopsy of the fetus, together with gross and histologic examination of the umbilical cord, placenta, and membranes, fails to demonstrate an adequate cause of death”.

In 2002, Matturri et al. (24) reported the first anatomo-pathological evidence of hypoplasia or agenesis of the arcuate nucleus in unexpected stillborns, in a similar manner than that detected in SIDS victims. Since then, additional anatomo-pathological studies confirmed similar findings in the autonomic nervous and cardiac conduction systems in both SIDS and SIUDS victims (2, 9, 11).

In 2006, the Italian Law no. 31 (10), “Regulations for diagnostic postmortem investigation in victims of SIDS and unexpected fetal death”, was introduced, imposing common rules for the referral of cases and postmortem procedures equally in victims of SIDS and unexpected sudden fetal death starting from the 25th gestational week.

In 2009, the then US Senator Barack Obama (25) introduced the Preventing Stillbirth and SUID Act, which enhances public health activities related to understanding and preventing unexplained stillbirth and SUID.

In 2014, Matturri et al. (26) defined sudden intrauterine unexplained death syndrome (SIUDS) as “The sudden death during pregnancy that remains unexplained after an in-depth autopsy including examination of the placental disk, umbilical cord and membranes, detailed pregnancy history analysis and molecular and microbiological investigations”.

Sudden intrauterine unexplained/unexpected death syndrome, or unexpected stillbirth, is herein defined as “The late fetal death before the complete expulsion or removal of the fetus from the mother ≥25 weeks of gestation which is unexpected by history and is unexplained after review of the maternal clinical history and the performance of a general autopsy of the fetus, including examination of the placental disk, umbilical cord and membranes, and microbiological and genetic investigations”.

### Sudden Unexpected Infant and Perinatal Death

The acronym “SPUD” for sudden unexpected infant and perinatal death, which itself includes SIUDS and sudden neonatal unexpected death (SNUD) (2).

Perinatal mortality refers to death around the time of delivery and includes both fetal deaths (at least 20 weeks of gestation) and neonatal (early infant) deaths (27). Pathologically, SIDS and SIUDS can be included in the extended domain of perinatal-infant pathology, as a continuity of cardiac conduction system and autonomic nervous system findings have been detected without a clear separation between sudden unexpected perinatal and infant death. The in-depth postmortem examination is equally mandatory in every case of sudden unexpected infant and perinatal death (SPUD), which itself includes SIUDS and SNUD.

### Sudden Neonatal Unexpected Death

Sudden infant death syndrome’s pathology includes an extended domain of neonatal pathology, particularly if within the diagnosis of SIDS one wishes to enclose the acronym “SNUD” for sudden neonatal unexpected death, not definitely separable from the unifying concept of syndrome (2).

### SIDS–SIUDS Complex

The SIDS–SIUDS complex describes a multifactorial pathology likely consisting of congenital anomalies of the cardiac conduction system and of the autonomic nervous system, mainly cardiorespiratory, of the first upper-digestive pathways, and arousal, detected in both infants and fetuses dying suddenly and unexpectedly (2). Both, SIDS and SIUDS, could be attributed to multiple causes and characterized by the unifying etiological concept of “syndrome”, adopted to describe the SIDS–SIUDS complex as a frequent form of death in fetuses and infants.

Commonalities have been reported among cases of unexplained stillbirth and SIDS, i.e., dysfunction in limbic forebrain, hippocampus, and brainstem circuits, designated as the central homeostatic network and the triple risk model (22, 28, 29).

On the basis of these considerations, the herein presented new common definition of the SIDS–SIUDS complex is “The sudden death of a fetus after the 25th gestational week or infant under one year of age which is unexpected by history and remains unexplained after a thorough case investigation, including examination of the death scene, performance of a general autopsy and examination of the fetal adnexa”. Therefore, given that the general autopsy does not disclose any cause of death, a more in-depth histopathological analysis of the cardiac conduction system and autonomic nervous system by specialized pathologists is necessary.

### SIDS and SIUDS Gray Zone

In 1995, Gregerson et al. (30), in the Nordic SIDS study, described the SIDS borderline cases in which pre-existing congenital disorders or clinical symptoms and/or postmortem findings are not severe enough to explain the cause of death.

In 2001, Rognum (31) described the gray zone as a group of “in between” cases in which there are pathological findings, either morphologic or microbiologic, or information from the history or the circumstances of death, that are significant, but most likely not sufficient to explain death. This gray zone may also include neglect, abuse, and even murder.

In 2004, Krous et al. (21) in their definition of SIDS, did not mention a SIDS gray zone, and described as “unclassified sudden infant deaths” those cases that do not meet the criteria for a diagnosis of SIDS with equivocal alternative diagnoses of natural or unnatural conditions. They anticipated that their definitions of SIDS would have been modified to accommodate new understandings of SIDS and sudden infant death.

In 2007, Matturri et al. (32) described nine fetuses that died suddenly and unexpectedly with concomitant abnormalities of the fetal adnexa, classified as SIUD gray zone. These cases presented with cardiac conduction and brainstem lesions in association with choriamnionitis (seven cases), abnormally short umbilical cord (one case), and placental infection by parvovirus (one case), which alone might not have accounted for the sudden death.

Sudden infant death syndrome and SIUDS are classified as borderline or gray zone when the sudden unexpected death is concomitant to a coexistent disease, such as mild to moderate bronchus-pneumonic infection or chorionamnionitis, itself not enough to cause death, acting as a triggering phenomenon in a vulnerable infant or fetus (2, 9, 28, 33).

In the SIDS/SIUDS gray zone the infants/fetuses are vulnerable due to pre-existing nervous, autonomic, or cardiac conduction alterations, such as hypoplasia of the arcuate nucleus or a Mahaim fiber, which are plausible bases for the diagnosis of SIDS/SIUDS, and with the lethal event being triggered by the concomitant pathology.

Following these considerations, the SIDS/SIUDS gray zone is now defined as: “The infant/late fetal death ≥25 weeks of gestation which is unexpected by history and is unexplained after review of the clinical history and the performance of a general autopsy which includes examination of the fetal adnexa, as well as microbiological, and genetic investigations, which occurs with another event, acting as a triggering phenomenon itself not enough to cause death, in a vulnerable infant/fetus”.

## Concluding Remarks

Congenital abnormalities, likely morphological substrates for SIDS and SIUDS, were frequently detected, mainly represented by alterations of the cardiac conduction system, such as accessory pathways and abnormal resorptive degeneration, and hypoplasia/agenesis of the vital brainstem structures (2, 9, 11, 24).

Current SIDS–SIUDS research is attempting to integrate the anatomo-pathological findings with the genetic and environmental substrates. Any case of suspected SIDS or SIUDS should be submitted to an in-depth postmortem examination, particularly focused on the investigation of the cardiac conduction system and brainstem on serial sections, along with toxicological–environmental and genetic investigations.

## Author Contributions

GO has primary responsability for development of the concept and design, writing, drafting, and revising the manuscript.

## Conflict of Interest Statement

The author declares that she has no conflicts of interest, financial, or otherwise, to declare.
